# Formal tobacco-control training and thirdhand smoke counseling practices among pediatric nurses in China: a cross-sectional study of cognitive and implementation factors

**DOI:** 10.3389/fpubh.2026.1781485

**Published:** 2026-07-08

**Authors:** Li Zhang, Yingji Tang, Yang Yang, Mingyue Chen

**Affiliations:** 1 Department of Stomatology, Taihe Hospital, Hubei University of Medicine, Shiyan, Hubei, China; 2 Nursing Department, The Third Xiangya Hospital, Central South University, Changsha, Hunan, China; 3 Respiratory and Critical Care Medicine Department, The Third Xiangya Hospital of Central South University, Changsha, Hunan, China

**Keywords:** China, health equity, implementation science, pediatric nurses, thirdhand smoke, tobacco control

## Abstract

**Background:**

Thirdhand smoke (THS) exposure poses significant health risks to children, yet pediatric healthcare providers’ capacity to deliver evidence-based counseling remains poorly characterized, particularly in high-burden settings. This study examined THS counseling practices among pediatric nurses in China, with particular emphasis on the relative contributions of formal tobacco-control training, cognitive factors, and implementation context.

**Methods:**

We conducted a cross-sectional survey of pediatric nurses at The Third Xiangya Hospital of Central South University, Changsha, China. Data were collected between February 2022 and October 2025 (response rate: 83.7%). Participants completed validated instruments assessing THS knowledge, attitudes, self-efficacy, counseling practices, implementation factors, and clinical performance using standardized vignettes. Sequential multivariable linear regression was used to identify independent predictors of counseling practices.

**Results:**

Among 633 pediatric nurses (mean age 32.1 ± 5.7 years; 91.6% female), 248 (39.2%) had received formal tobacco-control training in the preceding 24 months. Training was the strongest correlate of counseling practices (*β* = 2.23; 95% CI, 1.56–2.89; *p* < 0.001; ΔR^2^ = 0.068), while knowledge, attitudes, and self-efficacy added negligible explanatory power (ΔR^2^ = 0.004). Yet absolute practice rates remained suboptimal even among trained nurses: only 51.6% consistently explained THS concepts and 44.4% offered cessation referrals. Only 34.4% correctly rejected the misconception that absence of odor indicates no THS exposure. Vignette scores declined from 20.16 ± 1.56 for healthy infant scenarios to 18.43 ± 1.76 for disadvantaged families (*p* < 0.001), despite their higher household smoking burden. High facilitator availability (74–80%) did not predict practice scores, pointing to workflow integration deficits rather than resource shortfalls.

**Conclusion:**

Training is associated with more frequent THS counseling, yet misconceptions persist and disadvantaged families receive lower-quality care. Prospective trials are needed to test whether skills-based training redesign and workflow-embedded decision supports can translate this association into equitable improvements in child health protection.

## 1 Introduction

Childhood exposure to tobacco smoke remains a primary preventable driver of global morbidity. Beyond the well-established harms of secondhand smoke (SHS), robust evidence identifies thirdhand smoke (THS) as a distinct environmental hazard—residual tobacco pollutants that adsorb to indoor surfaces, dust, clothing, and skin, persisting long after smoking cessation (1–3). With more than 1.3 billion tobacco users worldwide, children face disproportionate exposure within homes and caregiving settings, particularly in low- and middle-income countries where private enforcement of smoke-free norms lags behind public policy (1, 4). THS differs fundamentally from SHS: rather than dissipating, it comprises a complex mixture of nicotine, tobacco-specific nitrosamines, polycyclic aromatic hydrocarbons, and volatile organic compounds that undergo chemical aging and re-emission. This transformation creates prolonged exposure pathways that are particularly hazardous for infants and young children (1, 5, 6). Consequently, THS persists as a uniquely pervasive threat with critical implications for pediatric health equity and clinical prevention strategies.

These findings underscore a clinical imperative: pediatric providers must recognize THS as a persistent exposure requiring proactive counseling. Advice contingent on visible smoke or odor is insufficient (7, 8). Animal models provide compelling evidence that early-life THS exposure induces oxidative stress, impaired lung development, metabolic dysregulation, and immune alterations, independent of SHS co-exposure (9, 10). In clinical settings, biomarkers of THS exposure, such as cotinine and tobacco-related nitrosamine metabolites, are detectable in children despite the absence of household smoking at the time of assessment (11, 12). Collectively, these findings establish THS as a biologically active exposure capable of exerting clinically meaningful effects during critical developmental windows (1, 13, 14).

Population-based studies link THS-contaminated environments to measurable adverse outcomes, including elevated rates of respiratory symptoms, asthma exacerbations, neurobehavioral impairment, and systemic inflammation (15, 16). Infants and toddlers face heightened vulnerability through frequent hand-to-mouth contact, proximity to contaminated surfaces, elevated respiratory rates relative to body size, and immature metabolic detoxification pathways (17, 18). The absence of sensory cues, such as odor, often leads caregivers to underestimate risk, reinforcing persistent misconceptions that smoking near windows, on balconies, or in the absence of children sufficiently protects against exposure (19, 20). Correcting these misconceptions requires healthcare professionals capable of translating THS science into actionable parental counseling.

Pediatric nurses represent a frontline workforce for tobacco exposure prevention through their roles in health education, risk assessment, and anticipatory guidance. Their frequent interactions with families during well-child visits, acute illness episodes, and chronic disease follow-up create iterative opportunities to evaluate household smoking behaviors and promote smoke-free environments (21–23). Despite generally positive attitudes toward tobacco control, nurses’ knowledge of THS mechanisms and confidence in counseling remain variable and often suboptimal (24, 25). Evidence from high-income settings demonstrates that structured tobacco-control training improves knowledge precision, counseling rates, and intervention documentation, although substantial deficits persist even among trained providers (26, 27). Notably, existing literature predominantly examines physicians or heterogeneous healthcare samples, yielding limited insight into pediatric nurses’ THS-specific competencies (28).

China presents a context of particular urgency for THS prevention, accounting for the world’s largest smoking population—an estimated 300 million adults—and pervasive household tobacco use despite expanding national tobacco-control legislation (29–31). National surveillance data indicate that over half of Chinese children experience household tobacco smoke exposure, with THS contamination documented even in homes reporting smoke-free policies (29, 32, 33). Cultural acceptance of indoor smoking, multigenerational households, and minimal public THS awareness compound exposure risks, disproportionately affecting infants and economically vulnerable populations (33–35).

However, empirical characterization of Chinese healthcare providers’ THS knowledge remains sparse. Available studies have focused on physician or general nurse awareness of SHS, typically emphasizing policy familiarity over mechanistic comprehension or behavioral counseling (36, 37). Few studies have explicitly assessed THS-related knowledge, misconceptions, or counseling behaviors, and none, to our knowledge, have comprehensively examined pediatric nurses across clinical settings using validated knowledge items, practice measures, and implementation context variables (38, 39). Crucially, whether formal tobacco-control training produces measurable improvements in THS counseling within authentic pediatric clinical scenarios remains an unanswered empirical question.

These knowledge gaps carry substantial clinical consequences. Inadequate THS literacy and unstructured counseling approaches limit nurses’ capacity to dispel parental misconceptions, promote comprehensive smoke-free environments, and interrupt persistent exposure during developmentally critical periods (40, 41). Determining whether training effects operate independently of provider attitudes, clinical experience, and institutional factors is essential for developing evidence-based, scalable interventions suited to resource-limited health systems (42). For China specifically, these questions are central to strengthening pediatric preventive services and narrowing tobacco-related health inequities.

To address these needs, we conducted a single-center investigation among Chinese pediatric nurses to characterize THS knowledge, attitudes, counseling behaviors, and clinical performance. Study objectives were to: (1) characterize the distribution and types of THS-related knowledge accuracy and misconceptions, without presuming the direction of findings; (2) evaluate the association between formal tobacco-control training, policy awareness, and evidence-based counseling practices; and (3) assess training effects on standardized vignette-based counseling performance across representative pediatric scenarios—including healthy infant supervision, pediatric asthma management, and care for socioeconomically disadvantaged families.

## 2 Methods

### 2.1 Study design and setting

This study employed a single-center, cross-sectional survey design to examine pediatric nurses’ knowledge, attitudes, and counseling practices regarding thirdhand smoke (THS) exposure in children. Data were collected between February 2022 and October 2025 at The Third Xiangya Hospital of Central South University (Changsha, Hunan, China), a tertiary-level maternal and child health hospital that provides comprehensive pediatric services across multiple specialized units. The institution includes general pediatrics, neonatal intensive care (NICU), pediatric intensive care (PICU), outpatient pediatrics, and emergency care departments. The hospital serves a predominantly urban population while also receiving referrals from peri-urban and rural catchment areas, thereby allowing assessment of practice patterns across diverse clinical settings and patient populations.

### 2.2 Study population and recruitment

Eligible participants were registered pediatric nurses actively involved in direct patient care at the time of the study. Inclusion criteria comprised: (1) current employment in pediatric clinical units including general pediatrics, NICU, PICU, outpatient pediatrics, or pediatric emergency departments; (2) active provision of routine or acute care to infants and children as part of regular clinical duties; and (3) at least 6 months of clinical experience at the study institution to ensure adequate familiarity with institutional workflows, patient populations, and counseling practices. Nurses on extended leave, those in exclusively administrative roles without direct patient contact, and those working in non-pediatric units were excluded. All eligible nurses were invited to participate through departmental briefings and electronic notifications. Of 756 eligible nurses, 633 completed the survey in its entirety (response rate: 83.7%), with non-respondents primarily citing workload constraints (*n* = 89) or declining participation without stated reason (*n* = 34). Non-respondents did not differ significantly from respondents in age, gender, or unit distribution based on administrative records (all *p* > 0.10).

### 2.3 Data collection

Data were collected using a structured, self-administered questionnaire developed from established THS literature, pediatric tobacco-control frameworks, and validated healthcare provider surveys (43). The instrument underwent pilot testing for clarity and face validity. Surveys were administered in paper or secure electronic format during scheduled work periods, with completed surveys collected via sealed envelopes or encrypted electronic submission (44).

### 2.4 Outcome measures

#### 2.4.1 Sociodemographic and professional characteristics

Participants reported age (continuous, years), gender (female, male, or prefer not to answer), highest educational attainment (diploma, bachelor’s, master’s, doctorate), years of pediatric nursing practice (<5, 5–9, 10–19, ≥20 years), and primary practice setting (general pediatrics, NICU, PICU, outpatient, emergency). For regression analyses, gender was coded as female versus male/prefer not to answer, education as bachelor degree or higher versus diploma, years of practice ordinally as 1–4, and practice setting as urban versus peri-urban/rural. The primary exposure was formal tobacco-control training, assessed as a binary variable indicating receipt of formal tobacco-control training within the 24 months immediately preceding survey completion (yes/no). Given the 45-month data collection window (February 2022–October 2025), training exposure windows ranged from February 2020–February 2022 for early participants to October 2023–October 2025 for later participants.

Training was assessed as a binary self-report variable (trained/untrained in the preceding 24 months), and content, duration, and pedagogy were not measured systematically. Therefore, the observed association with counseling practices cannot be attributed to any specific training component, and the exposure likely reflects heterogeneous training experiences. Institutional records suggest training usually involved 2–8-h workshops, but content and delivery likely varied across departments and time periods. This heterogeneity limits precision and prevents identification of the components associated with better reported practices. Accordingly, the Discussion’s training recommendations should be interpreted as literature-informed proposals rather than direct inferences from these data.

#### 2.4.2 Policy awareness

Awareness of institutional tobacco-control and smoke-free policies was measured using a single categorical item with three mutually exclusive response options. Participants indicated whether they were aware of existing institutional policies, unsure about policy existence or content, or not aware of any such policies. Awareness of institutional tobacco-control and smoke-free policies was measured using a single categorical item with three mutually exclusive response options: aware, unsure, or not aware. For all bivariate comparisons, the three-level distribution is reported in Table 1. For regression analyses, policy awareness was operationalized as a binary variable contrasting nurses who were definitively aware of institutional policies versus those who were either unsure or unaware, consistent with the primary analytical question of whether policy recognition was associated with counseling practices. This single operationalization applies consistently across all regression models reported in Table 2.

**Table 1:** Characteristics of pediatric nurses stratified by training status, practice setting, and education (*N* = 633).

Characteristic	Overall	Trained	Untrained	Urban	Rural	*p* value
	No. (%)	No. (%)	No. (%)	No. (%)	No. (%)	
Age, mean ± SD, y	32.1 ± 5.7	32.6 ± 5.6	31.8 ± 5.8	32.3 ± 5.6	31.2 ± 6.1	0.041
Woman, No. (%)	580 (91.6)	232 (93.5)	348 (90.4)	366 (92.0)	76 (89.4)	0.127
Man, No. (%)	47 (7.4)	15 (6.0)	32 (8.3)	28 (7.0)	9 (10.6)	
Diploma, No. (%)	81 (12.8)	20 (8.1)	61 (15.8)	45 (11.3)	15 (17.6)	0.002
Bachelor, No. (%)	435 (68.7)	178 (71.8)	257 (66.8)	277 (69.6)	54 (63.5)	
Master, No. (%)	102 (16.1)	45 (18.1)	57 (14.8)	67 (16.8)	13 (15.3)	
Doctorate, No. (%)	15 (2.4)	5 (2.0)	10 (2.6)	9 (2.3)	3 (3.5)	
General pediatrics, No. (%)	218 (34.4)	91 (36.7)	127 (33.0)	143 (35.9)	26 (30.6)	0.156
NICU, No. (%)	116 (18.3)	45 (18.1)	71 (18.4)	76 (19.1)	12 (14.1)	
PICU, No. (%)	95 (15.0)	34 (13.7)	61 (15.8)	62 (15.6)	11 (12.9)	
Outpatient pediatrics, No. (%)	97 (15.3)	43 (17.3)	54 (14.0)	55 (13.8)	17 (20.0)	
Emergency, No. (%)	84 (13.3)	28 (11.3)	56 (14.5)	49 (12.3)	15 (17.6)	
Experience <5 years, No. (%)	188 (29.7)	63 (25.4)	125 (32.5)	114 (28.6)	30 (35.3)	0.041
Experience 5–9 years, No. (%)	208 (32.9)	86 (34.7)	122 (31.7)	131 (32.9)	27 (31.8)	
Experience 10–19 years, No. (%)	175 (27.6)	75 (30.2)	100 (26.0)	113 (28.4)	20 (23.5)	
Experience ≥20 years, No. (%)	62 (9.8)	24 (9.7)	38 (9.9)	40 (10.1)	8 (9.4)	
Policy awareness: Yes, No. (%)	302 (47.7)	143 (57.7)	159 (41.3)	199 (50.0)	35 (41.2)	<0.001
Policy awareness: Unsure, No. (%)	200 (31.6)	69 (27.8)	131 (34.0)	123 (30.9)	28 (32.9)	
Policy awareness: No, No. (%)	131 (20.7)	36 (14.5)	95 (24.7)	76 (19.1)	22 (25.9)	

Data are No. (%) unless otherwise stated. Trained: received formal tobacco-control training in the 24 months preceding survey completion. Untrained: no such training in that period. *p* values compare trained vs untrained nurses; χ^2^ for categorical variables, Mann–Whitney U for continuous variables. NICU, neonatal intensive care unit; PICU, pediatric intensive care unit. Urban: hospital within Changsha city proper (population >1 million). Rural: satellite facility serving counties or townships (population <500,000).

**Table 2:** Sequential regression models predicting thirdhand smoke counseling practice score (*N* = 633).

Variable	Model 1	*p*	Model 2	*p*	Model 3	*p*	Model 4	*p*
	β (95% CI)		β (95% CI)		β (95% CI)		β (95% CI)	
Age, y	−0.01 (−0.08 to 0.05)	0.691	−0.02 (−0.08 to 0.05)	0.627	−0.01 (−0.07 to 0.05)	0.728	−0.02 (−0.07 to 0.04)	0.599
Female gender	0.23 (−1.12 to 1.59)	0.733	0.26 (−1.08 to 1.60)	0.706	0.30 (−1.02 to 1.62)	0.655	0.36 (−0.95 to 1.66)	0.594
Bachelor degree or higher	0.08 (−0.87 to 1.02)	0.871	−0.11 (−1.04 to 0.82)	0.814	−0.15 (−1.07 to 0.77)	0.752	−0.19 (−1.15 to 0.77)	0.701
Years of practice	0.04 (−0.32 to 0.40)	0.827	0.06 (−0.30 to 0.42)	0.734	0.07 (−0.28 to 0.42)	0.691	0.07 (−0.26 to 0.40)	0.672
Urban setting	−0.07 (−0.73 to 0.59)	0.838	−0.03 (−0.69 to 0.62)	0.921	−0.06 (−0.71 to 0.59)	0.854	−0.11 (−0.77 to 0.56)	0.757
Tobacco-control training	—	—	2.31 (1.65 to 2.97)	<0.001	2.28 (1.63 to 2.93)	<0.001	2.23 (1.56 to 2.89)	<0.001
Knowledge score (0–10)	—	—	—	—	0.14 (−0.08 to 0.36)	0.217	0.14 (−0.08 to 0.36)	0.216
Attitude score (8–40)	—	—	—	—	0.05 (−0.08 to 0.18)	0.447	0.05 (−0.08 to 0.19)	0.431
Self-efficacy score (0–60)	—	—	—	—	0.00 (−0.08 to 0.08)	0.991	0.00 (−0.08 to 0.08)	0.99
Barrier score (4–20)	—	—	—	—	—	—	−0.11 (−0.28 to 0.06)	0.188
Facilitator score (4–20)	—	—	—	—	—	—	0.10 (−0.09 to 0.29)	0.297
Leadership support (1–5)	—	—	—	—	—	—	−0.03 (−0.37 to 0.31)	0.862
Aware of institutional policies	—	—	—	—	—	—	0.28 (−0.41 to 0.97)	0.427
Model R^2^	0.002		0.07		0.074		0.075	

Dependent variable: practice score (range, 10–50). β indicates regression coefficient; CI, confidence interval. Model 1 (Sociodemographic): Adjusted for age, gender, education, years of practice, and setting (R^2^ = 0.002; minimal variance explained). Model 2 (Training): Model 1 plus tobacco-control training (R^2^ = 0.070; ΔR^2^ = 0.068), indicating 6.8% additional variance explained. Model 3 (Cognitive): Model 2 plus knowledge (0–10), attitude (8-40), and self-efficacy (0–60) scores (R^2^ = 0.074; ΔR^2^ = 0.004), showing negligible improvement. Model 4 (Full Implementation): Model 3 plus barriers/facilitators (4-20), leadership support, and policy awareness (R^2^ = 0.075; ΔR^2^ = 0.001), indicating no significant prediction enhancement.

#### 2.4.3 Thirdhand smoke knowledge

THS knowledge was assessed using a 10-item scale designed to capture core domains of THS science relevant to pediatric nursing practice. The knowledge items were constructed to reflect consensus domains in the thirdhand smoke literature and were designed to assess applied clinical understanding rather than factual recall alone. The scale assessed THS residue persistence, pollutant re-emission, pediatric exposure pathways (e.g., ingestion, inhalation), infant vulnerability, and associated health risks (45). However, the scale deliberately included three items framed as common misconceptions requiring identification of false statements as the correct response. These misconception items stated that absence of tobacco odor means no THS is present, that smoking on a balcony or near an open window fully prevents indoor exposure, and that routine cleaning always eliminates THS from the environment. Correct identification of these as false statements was necessary for full knowledge credit.

Each of the 10 items was scored dichotomously, with correct responses receiving 1 point and incorrect responses or “do not know” selections receiving 0 points. Composite knowledge scores were calculated by summing correct responses (range: 0–10), with higher scores indicating greater accuracy. Internal consistency was poor (Cronbach’s *α* = 0.060), suggesting items measure multiple independent factual domains rather than a unified construct, warranting cautious interpretation of composite scores and preference for item-level analysis. This level of internal consistency is below acceptable psychometric thresholds for composite score use. Accordingly, the knowledge composite is retained in regression models only as a covariate and its coefficient should be interpreted with caution; attenuation bias due to measurement error may cause the true knowledge–practice association to be underestimated. All primary knowledge findings are reported and interpreted at the item level. Because the 10 items were designed to span distinct, non-redundant content domains—persistence, re-emission, exposure pathways, infant vulnerability, health effects, and misconception identification—statistical covariance across items is neither expected nor desirable from a content-validity standpoint. Nevertheless, poor internal consistency precludes valid interpretation of the composite knowledge score as a unidimensional construct. Accordingly, all knowledge findings are reported and interpreted at the item level, and composite knowledge scores are used only as a covariate in regression models with the caveat that this measure carries limited psychometric coherence. This limitation is discussed further in the limitations section.

#### 2.4.4 Attitudes toward THS counseling

Attitudes regarding the importance and appropriateness of THS counseling in pediatric nursing practice were measured using an 8-item Likert-scale instrument. The attitude scale assessed multiple dimensions of nurses’ beliefs including their perceptions of the relevance and importance of THS to child health outcomes, their understanding of the professional role of nurses in tobacco exposure prevention, their views on the effectiveness of nursing counseling in changing parental smoking behaviors, and their beliefs about the acceptability and appropriateness of discussing household smoking behaviors with caregivers during routine pediatric encounters (46, 47).

Each item was rated on a 5-point Likert scale (1 = strongly disagree to 5 = strongly agree), with all items worded such that higher ratings reflected more favorable or supportive attitudes toward THS counseling as a nursing responsibility. Composite attitude scores were calculated as the sum of ratings across all eight items (range: 8–40), with higher scores reflecting more favorable dispositions toward engaging in THS counseling activities with parents and caregivers. Internal consistency was acceptable (Cronbach’s *α* = 0.78), indicating a reasonably coherent underlying construct. Internal consistency was acceptable (Cronbach’s α = 0.78), indicating a reasonably coherent underlying construct.

#### 2.4.5 Self-efficacy for counseling

Self-efficacy, defined as nurses’ confidence in their ability to successfully perform specific THS counseling behaviors, was assessed using a 6-item scale. The scale evaluated perceived competence across key counseling components that align with evidence-based tobacco control recommendations for pediatric healthcare settings (48). Questions addressed confidence in six key areas: explaining THS concepts, assessing risk, advising on mitigation, referring for cessation, documenting care, and tracking follow-up.

Each item employed an 11-point numerical rating scale ranging from 0 (not confident at all in performing the behavior) to 10 (extremely confident in one’s ability to execute the behavior successfully). This extended response scale was chosen to provide greater sensitivity in detecting variations in confidence levels compared to traditional 5-point scales. Composite self-efficacy scores were calculated by summing item ratings (range: 0–60), with higher scores reflecting greater perceived counseling competence and readiness to engage in comprehensive THS counseling. Internal consistency was good (Cronbach’s *α* = 0.85), supporting use of the composite score.

#### 2.4.6 Counseling practices

The primary outcome variable was routine THS counseling practices, measured using a 10-item practice scale reflecting evidence-based pediatric tobacco-control behaviors recommended by professional guidelines and tobacco control frameworks (49). The scale captured the full spectrum of counseling activities that nurses might undertake during patient encounters. Practice items included screening for smoking behaviors and rules, explaining the inadequacy of spatial separation, advising on THS mitigation and smoke-free environments, providing cessation referrals, utilizing educational materials, and documenting follow-up.

Each practice item was rated using a 5-point frequency scale with response options corresponding to never performing the behavior (scored as 1), rarely performing it (scored as 2), sometimes performing it (scored as 3), often performing it (scored as 4), and always performing it (scored as 5). For descriptive analyses examining the prevalence of optimal counseling behaviors, responses of “often” or “always” (scores 4–5) were categorized as representing optimal practice for that particular counseling component. Composite practice scores were calculated by summing frequency ratings across all 10 items (range: 10–50), with higher scores reflecting more comprehensive counseling. Participants with >2 missing practice items (*n* = 0) were excluded from composite score analyses. For participants with 1–2 missing items (*n* = 18), missing values were imputed using the respondent’s mean score across completed items. Composite practice scores were calculated by summing frequency ratings across all 10 items (range: 10–50), with higher scores reflecting more comprehensive THS counseling. Internal consistency was low (Cronbach’s *α* = 0.249), suggesting these represent distinct behaviors rather than a unified syndrome. This composite score served as the primary dependent variable as a behavioral breadth index—reflecting the number of counseling domains engaged rather than a unidimensional latent trait—with the explicit caveat that low reliability attenuates regression coefficients toward the null and reduces precision of effect estimates. Readers should interpret the magnitude of regression associations with this limitation in mind, as true associations may be larger than observed. All participants (*n* = 633) completed knowledge, attitude, and self-efficacy scales in their entirety. Eighteen participants (2.8%) had 1–2 missing practice items requiring mean imputation.

#### 2.4.7 Barriers and facilitators to implementation

Perceived barriers to implementing THS counseling in routine clinical practice were assessed using a 4-item scale addressing common obstacles identified in prior tobacco control implementation research (50). Barrier items evaluated whether nurses felt they had insufficient time during clinical encounters to address THS counseling adequately, whether they perceived a lack of training or educational materials necessary to counsel effectively, whether they experienced fear or discomfort about offending parents by discussing household smoking behaviors, and whether they perceived an absence of clear institutional protocols or guidelines for THS screening and counseling.

Perceived facilitators of THS counseling implementation were measured using a separate parallel 4-item scale assessing supportive factors that could enable or enhance counseling behaviors. Facilitator items evaluated whether nurses had access to standardized counseling scripts or talking points to guide THS discussions, whether clear referral pathways existed to connect families with cessation resources, whether institutional leadership emphasized tobacco control as a priority area, and whether peer champions or role models existed who could provide support and demonstrate effective counseling approaches.

All barrier and facilitator items employed 5-point Likert scales (1 = strongly disagree to 5 = strongly agree). For barriers, higher ratings reflected greater perceived obstacles to implementation, whereas for facilitators, higher ratings reflected stronger perceived support. Separate composite scores for barriers and facilitators were calculated by summing ratings of their respective 4 items (range: 4–20 each). For descriptive reporting of individual items, high endorsement was operationally defined as Likert ratings of 4–5. Internal consistency was assessed using Cronbach’s alpha for both scales. The barrier scale demonstrated acceptable internal consistency (Cronbach’s *α* = 0.71) and the facilitator scale demonstrated acceptable internal consistency (Cronbach’s *α* = 0.76), supporting the use of composite scores for both implementation context measures. For reference, internal consistency across all six scales used in this study was as follows: knowledge (*α* = 0.060, poor; item-level analysis preferred), attitudes (α = 0.78, acceptable), self-efficacy (α = 0.85, good), counseling practices (α = 0.249, poor; composite used as behavioral breadth index), barriers (α = 0.71, acceptable), and facilitators (α = 0.76, acceptable).

#### 2.4.8 Leadership support and system-level factors

Leadership support was measured as a single-item continuous variable (1 = minimal support to 5 = strong support), analyzed separately from the facilitator scale. Four binary items assessed system-level factors: presence of routine screening protocols, electronic medical record documentation requirements, accessible referral resources, and inclusion in competency/quality improvement metrics (yes/no for each).

#### 2.4.9 Clinical vignette assessment

Three standardized clinical vignettes represented common pediatric scenarios: a healthy infant at routine well-child visit, a child with asthma exacerbation, and a socioeconomically disadvantaged family. For each vignette, participants rated likelihood of performing five counseling components: explaining THS, assessing home/car smoking, providing mitigation advice, offering cessation referrals, and documenting counseling. Components were rated on a 5-point scale (1 = would definitely not do to 5 = would definitely do), with high performance defined as ratings ≥4. Component scores were analyzed individually and summed within each vignette to generate composite scenario scores (range: 5–25), with higher scores reflecting greater likelihood of comprehensive counseling. Performance was compared across scenarios and stratified by training status and other characteristics (51).

### 2.5 Statistical analysis

Descriptive statistics summarized all variables. Categorical variables were reported as frequencies and percentages; continuous variables as means with standard deviations for normally distributed data or medians with interquartile ranges for skewed distributions. Bivariate analyses compared trained and untrained nurses. Chi-square tests assessed categorical outcomes, while Mann–Whitney U tests were employed for continuous variables given non-normal distributions of several scale-based measures. Training effect sizes for vignette components were calculated as mean differences between groups. Two-sided *p* values <0.05 were considered significant. Sequential multivariable linear regression identified independent predictors of composite counseling practice scores. Variables were entered in four theoretically motivated blocks. Model 1 included sociodemographic factors: age (continuous, years), gender (binary: female vs. male/prefer not to answer), education (binary: bachelor or higher vs. diploma), years of practice (ordinal: 1 = <5 years, 2 = 5–9 years, 3 = 10–19 years, 4 = ≥20 years), and practice setting (binary: urban vs. peri-urban/rural). Practice settings were classified as urban (hospitals located within Changsha city proper with population >1 million) versus peri-urban/rural (satellite facilities serving counties or townships with populations <500,000) based on institutional location and primary catchment area demographics. Model 2 added tobacco-control training status (binary: trained in past 24 months vs. untrained). Model 3 incorporated cognitive factors: knowledge score (continuous, 0–10), attitude score (continuous, 8–40), and self-efficacy score (continuous, 0–60). Model 4 added implementation context: barrier score (continuous, 4–20), facilitator score (continuous, 4–20), leadership support (continuous, 1–5), and policy awareness (binary: aware vs. unaware/unsure).

For each model, standardized regression coefficients (*β*) with 95% confidence intervals quantified associations. Model fit was evaluated using R-squared, with changes in R-squared (ΔR^2^) assessing incremental explanatory power of each variable block. Model diagnostics indicated no major violations of linear regression assumptions. Given the large number of statistical comparisons conducted (>100 tests across knowledge items, practice behaviors, vignette components, and subgroup analyses), we acknowledge substantial inflation of Type I error risk. However, readers should interpret *p*-values near 0.05 with appropriate caution, particularly for secondary analyses. We emphasize effect sizes and patterns of findings rather than isolated *p*-values. All analyses were conducted using R statistical software (version 4.4.2) with significance defined as *p* < 0.05.

## 3 Results

### 3.1 Participant characteristics

A total of 633 pediatric nurses completed the survey, comprising 248 (39.2%) who had received formal tobacco-control training within the preceding 24 months and 385 (60.8%) who had not. The mean age was 32.1 ± 5.7 years, with 91.6% identifying as female. Educational attainment was predominantly at the bachelor level (68.7%), followed by master’s degree (16.1%), diploma (12.8%), and doctorate (2.4%). Trained nurses were slightly older than untrained nurses (32.6 ± 5.6 vs. 31.8 ± 5.8 years, *p* = 0.041), though the absolute difference was modest (0.8 years). Given the large sample size, this statistically significant difference may not represent a clinically meaningful age disparity. In addition, trained nurses demonstrated significantly higher educational attainment, with 71.8% holding bachelor’s degrees compared to 66.8% of untrained nurses, and 18.1% versus 14.8% holding master’s degrees (*p* = 0.002; Table 1).

**Figure 1:**
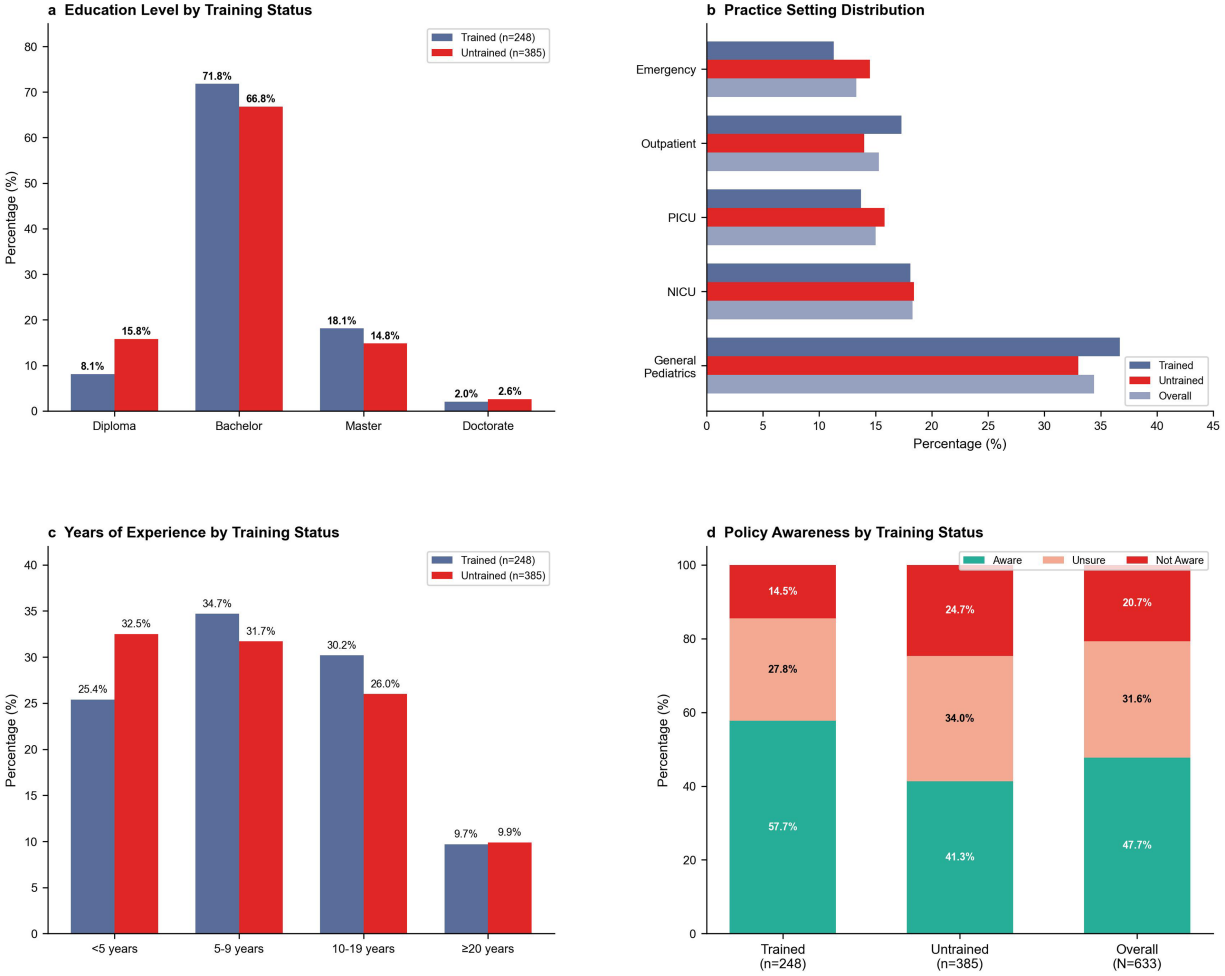
Characteristics of pediatric nurses by training status. Distribution of **(a)** educational attainment, **(b)** practice setting, **(c)** years of clinical experience, and **(d)** institutional tobacco-control policy awareness among trained and untrained pediatric nurses. Trained nurses demonstrated higher educational attainment and policy awareness compared to untrained nurses. NICU, neonatal intensive care unit; PICU, pediatric intensive care unit.

Years of nursing experience were broadly distributed, with 29.7% having less than 5 years, 32.9% having 5–9 years, 27.6% having 10–19 years, and 9.8% having 20 years or more of pediatric nursing practice. Trained nurses were more likely to have 10–19 years of experience (30.2% vs. 26.0%, *p* = 0.041). Practice settings included general pediatrics (34.4%), NICU (18.3%), PICU (15.0%), outpatient pediatrics (15.3%), and emergency departments (13.3%), with no significant differences in setting distribution between trained and untrained groups (*p* = 0.156; Figures 1a–c). Policy awareness differed substantially by training status. Among trained nurses, 57.7% were aware of institutional tobacco-control policies, compared to only 41.3% of untrained nurses (*p* < 0.001). Conversely, 24.7% of untrained nurses reported being unaware of such policies versus 14.5% of trained nurses (Figure 1d).

### 3.2 Thirdhand smoke knowledge

Overall THS knowledge varied considerably across items and was consistently higher among trained nurses. The best-recognized THS facts were that residues remain on surfaces after smoking stops (83.7% correct overall; trained: 88.3%, untrained: 80.8%, *p* = 0.011) and that THS can persist for weeks to months indoors (79.5% correct; trained: 83.9%, untrained: 76.6%, *p* = 0.027). Knowledge was strong regarding hand-to-mouth contact as an exposure route (72.7% correct; trained: 77.4%, untrained: 69.6%, *p* = 0.032) and re-emission of THS from surfaces into air (69.8% correct; trained: 75.4%, untrained: 66.2%, *p* = 0.015; Table 3).

**Table 3:** Thirdhand smoke knowledge by training status, practice setting, and experience level (*N* = 633).

Knowledge item	Overall	Trained	Untrained	Urban	Rural	<5 Years	≥10 Years	*p* value
	Correct	Correct	Correct	Correct	Correct	Correct	Correct	
	No. (%)	No. (%)	No. (%)	No. (%)	No. (%)	No. (%)	No. (%)	
Thirdhand smoke residues remain on surfaces after smoking stops	530 (83.7)	219 (88.3)	311 (80.8)	337 (84.7)	68 (80.0)	152 (80.9)	202 (85.2)	0.011
Thirdhand smoke can persist for weeks to months indoors	503 (79.5)	208 (83.9)	295 (76.6)	320 (80.4)	65 (76.5)	144 (76.6)	192 (81.0)	0.027
Absence of tobacco odor means no thirdhand smoke present (misconception)	218 (34.4)	102 (41.1)	116 (30.1)	144 (36.2)	26 (30.6)	61 (32.4)	85 (35.9)	0.005
Children exposed through hand-to-mouth contact with contaminated surfaces	460 (72.7)	192 (77.4)	268 (69.6)	295 (74.1)	58 (68.2)	133 (70.7)	175 (73.8)	0.032
Thirdhand smoke re-emits from surfaces into air over time	442 (69.8)	187 (75.4)	255 (66.2)	283 (71.1)	56 (65.9)	126 (67.0)	171 (72.2)	0.015
Handwashing and changing clothes reduce thirdhand smoke transfer	482 (76.1)	200 (80.6)	282 (73.2)	307 (77.1)	62 (72.9)	140 (74.5)	183 (77.2)	0.034
Infants and toddlers face higher risk than older children	455 (71.9)	188 (75.8)	267 (69.4)	291 (73.1)	59 (69.4)	132 (70.2)	173 (73.0)	0.089
Thirdhand smoke exposure causes health effects in children	395 (62.4)	169 (68.1)	226 (58.7)	254 (63.8)	50 (58.8)	112 (59.6)	153 (64.6)	0.017
Smoking on balcony or near window fully prevents indoor exposure (misconception)	231 (36.5)	103 (41.5)	128 (33.2)	150 (37.7)	29 (34.1)	65 (34.6)	89 (37.6)	0.034
Regular cleaning always eliminates thirdhand smoke from environment (misconception)	247 (39.0)	110 (44.4)	137 (35.6)	159 (39.9)	32 (37.6)	70 (37.2)	96 (40.5)	0.025

Correct responses indicate accurate understanding of thirdhand smoke biology, exposure mechanisms, and health effects. Three items marked as misconceptions require “False” response for accuracy. *p* values from χ^2^ tests comparing trained vs untrained nurses. Knowledge score calculated as sum of correct responses (range, 0–10); internal consistency Cronbach α = 0.060.

**Figure 2:**
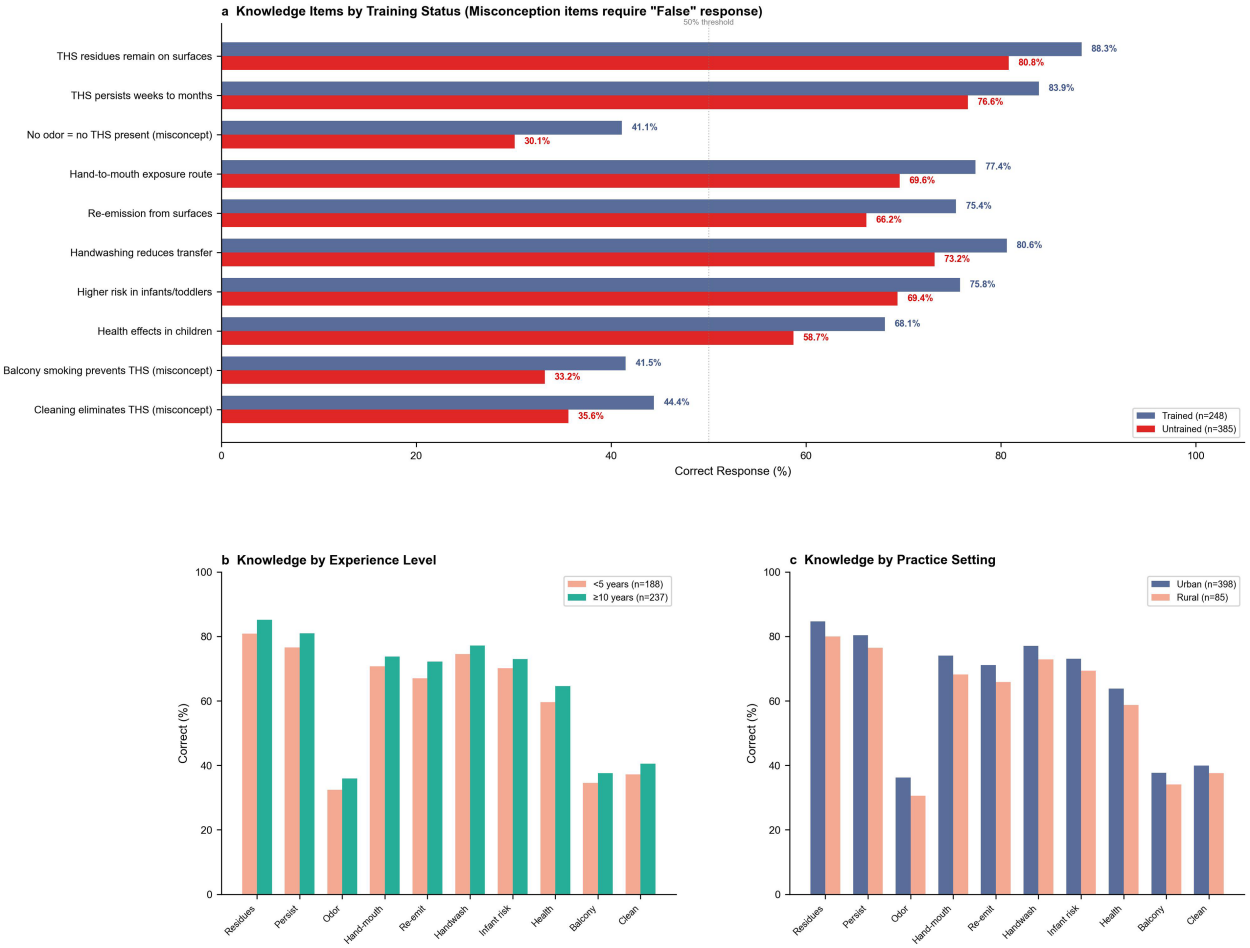
Thirdhand smoke knowledge by training status, setting, and experience. **(a)** Item-level knowledge accuracy for 10 THS knowledge items stratified by training status. Misconception items (marked with asterisk) required “False” responses for correct scoring. Dashed line indicates 90% accuracy threshold. **(b)** Knowledge performance by nursing experience level. **(c)** Knowledge performance by practice setting. Trained nurses consistently outperformed untrained nurses across all items, with largest gaps in misconception identification.

Critical knowledge gaps emerged around three misconceptions. Only 34.4% of nurses correctly identified that absence of tobacco odor does not mean THS is absent, with trained nurses performing better (41.1% vs. 30.1%, *p* = 0.005). Similarly, only 36.5% recognized that smoking on balconies or near windows does not fully prevent indoor exposure (trained: 41.5%, untrained: 33.2%, *p* = 0.034), and 39.0% correctly understood that regular cleaning does not always eliminate THS from environments (trained: 44.4%, untrained: 35.6%, *p* = 0.025; Figure 2a). Knowledge performance showed minimal variation by years of experience, with nurses having 10 or more years demonstrating only marginally higher accuracy on most items compared to those with less than 5 years of experience (Figure 2b). Urban versus rural practice setting yielded small differences, with urban nurses scoring 3–8 percentage points higher on most items (Figure 2c).

### 3.3 Counseling practices and training effects

Self-reported counseling practices were suboptimal across the sample, with significant training effects observed for all 10 measured behaviors. Among trained nurses, optimal practice rates (reporting “often” or “always” performing the behavior) ranged from 41.5% for recommending smoke-free environments to 51.6% for explaining THS concepts. For untrained nurses, rates were substantially lower, ranging from 32.7% for advising mitigation strategies to 39.0% for discussing the inadequacy of balcony smoking. Practice scores showed minimal variation by practice setting (urban: 40.7% optimal practice, rural: 37.6%, *p* = 0.156) or educational attainment (Bachelor+: 40.1%, Diploma: 37.8%, *p* = 0.284; Table 4).

**Table 4:** Thirdhand smoke counseling practices stratified by multiple nurse characteristics (*N* = 633).

Practice item	Mean ± SD	Overall	Trained	Untrained	Urban	Rural	Bachelor+	Diploma	*p* value
		Optimal	Optimal	Optimal	Optimal	Optimal	Optimal	Optimal	
		No. (%)	No. (%)	No. (%)	No. (%)	No. (%)	No. (%)	No. (%)	
Ask parents about household smoking or vaping	3.24 ± 1.21	252 (39.8)	118 (47.6)	134 (34.8)	162 (40.7)	32 (37.6)	222 (40.2)	30 (37.0)	0.001
Assess household smoke-free rules	3.19 ± 1.23	254 (40.1)	114 (46.0)	140 (36.4)	164 (41.2)	33 (38.8)	223 (40.4)	31 (38.3)	0.015
Discuss inadequacy of balcony or window smoking	3.29 ± 1.21	272 (43.0)	122 (49.2)	150 (39.0)	175 (44.0)	35 (41.2)	239 (43.3)	33 (40.7)	0.01
Explain thirdhand smoke concept and exposure pathways	3.30 ± 1.17	276 (43.6)	128 (51.6)	148 (38.4)	178 (44.7)	35 (41.2)	243 (44.0)	33 (40.7)	0.001
Advise on specific mitigation strategies	3.21 ± 1.17	232 (36.7)	106 (42.7)	126 (32.7)	150 (37.7)	30 (35.3)	204 (37.0)	28 (34.6)	0.009
Recommend smoke-free home and car	3.17 ± 1.10	233 (36.8)	103 (41.5)	130 (33.8)	149 (37.4)	30 (35.3)	205 (37.1)	28 (34.6)	0.047
Offer smoking cessation referrals	3.18 ± 1.22	242 (38.2)	110 (44.4)	132 (34.3)	155 (38.9)	31 (36.5)	213 (38.6)	29 (35.8)	0.01
Use educational materials about thirdhand smoke	3.22 ± 1.18	250 (39.5)	115 (46.4)	135 (35.1)	161 (40.5)	32 (37.6)	220 (39.9)	30 (37.0)	0.004
Document counseling in medical record	3.18 ± 1.20	244 (38.5)	109 (44.0)	135 (35.1)	157 (39.4)	31 (36.5)	215 (39.0)	29 (35.8)	0.024
Follow up on exposure reduction	3.18 ± 1.20	245 (38.7)	111 (44.8)	134 (34.8)	158 (39.7)	31 (36.5)	216 (39.1)	29 (35.8)	0.01

Practices rated on 5-point frequency scale: 1 = never, 2 = rarely, 3 = sometimes, 4 = often, 5 = always. Optimal practice defined as “always” or “often” performing the behavior. Bachelor+ includes Bachelor, Master, and Doctorate degrees. *p* values from χ^2^ tests comparing trained vs untrained nurses. Practice score calculated as sum of 10 items (range, 10–50); internal consistency Cronbach α = 0.249.

**Figure 3:**
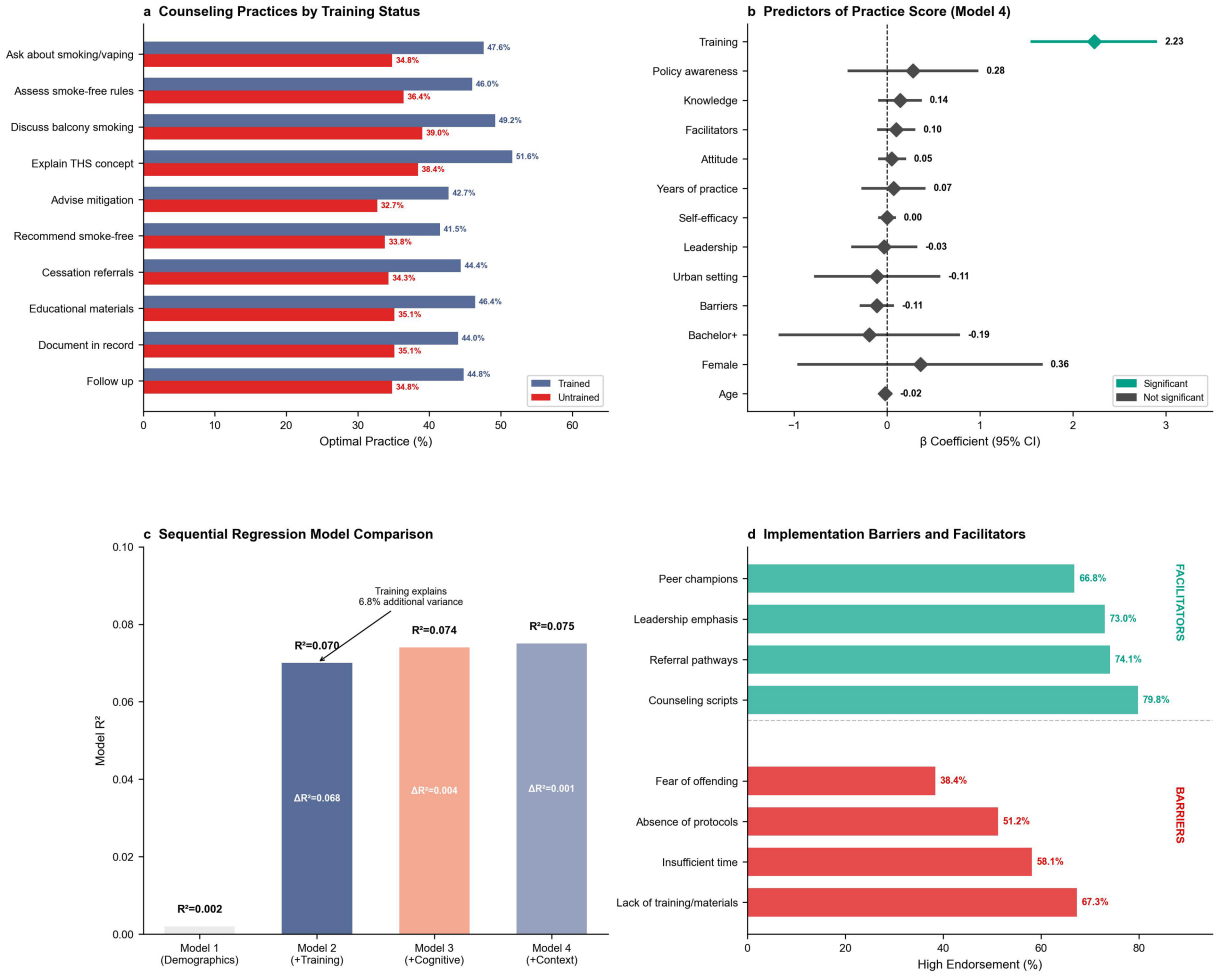
Thirdhand Smoke Counseling Practices, Predictors, and Implementation Factors. **(a)** Optimal practice rates for 10 counseling behaviors stratified by training status. **(b)** Forest plot showing predictors of practice scores from multivariable regression (full implementation model). Diamonds represent point estimates with 95% confidence intervals. Vertical dashed line indicates null effect. **(c)** Sequential regression model comparison showing incremental R^2^ contributions. ***p < 0.001. **(d)** Proportion of nurses endorsing each barrier (red) or facilitator (green) at rating ≥4 on a 5-point scale.

The largest training disparities appeared in asking about household smoking or vaping (trained: 47.6%, untrained: 34.8%, *p* = 0.001), explaining THS concepts (51.6% vs. 38.4%, *p* = 0.001), and offering cessation referrals (44.4% vs. 34.3%, *p* = 0.010). Trained nurses also demonstrated higher rates of assessing smoke-free rules (46.0% vs. 36.4%, *p* = 0.015), discussing inadequacy of balcony smoking (49.2% vs. 39.0%, *p* = 0.010), and using educational materials (46.4% vs. 35.1%, *p* = 0.004; Figure 3a). Mean composite practice scores reflected this training advantage. Trained nurses achieved significantly higher total practice scores across the 10-item scale, though absolute rates remained modest even among trained providers. Documentation practices and follow-up behaviors showed similar training gradients, with trained nurses reporting optimal documentation rates of 44.0% versus 35.1% for untrained nurses (*p* = 0.024).

### 3.4 Predictors of counseling practices

Sequential multivariable linear regression revealed that formal tobacco-control training was the dominant predictor of counseling practice scores, with minimal contribution from other factors. Model 1, including only sociodemographic and professional characteristics (age, gender, education, years of practice, practice setting), explained virtually no variance in practice scores (R^2^ = 0.002). Addition of training status in Model 2 resulted in substantial improvement (R^2^ = 0.070, ΔR^2^ = 0.068), with training associated with a *β* coefficient of 2.31 (95% CI: 1.65–2.97, *p* < 0.001; Table 2).

Model 3, incorporating cognitive factors (knowledge, attitudes, self-efficacy), added negligible explanatory power (R^2^ = 0.074, ΔR^2^ = 0.004). Neither knowledge score (*β* = 0.14, 95% CI: −0.08 to 0.36, *p* = 0.217), attitude score (*β* = 0.05, 95% CI: −0.08 to 0.18, *p* = 0.447), nor self-efficacy score (*β* = 0.00, 95% CI: −0.08 to 0.08, *p* = 0.991) demonstrated significant independent associations with practice when training was included in the model. Model 4, the full implementation model adding implementation factors (barriers, facilitators, leadership support, policy awareness), provided no further improvement (R^2^ = 0.075, ΔR^2^ = 0.001). None of the implementation variables achieved statistical significance: barriers (*β* = −0.11, *p* = 0.188), facilitators (*β* = 0.10, *p* = 0.297), leadership support (*β* = −0.03, *p* = 0.862), or policy awareness (*β* = 0.28, *p* = 0.427). The training coefficient remained stable and significant across all models (Model 4: *β* = 2.23, 95% CI: 1.56–2.89, *p* < 0.001), indicating robustness to adjustment (Figures 3b,c).

### 3.5 Clinical vignette performance

**Figure 4:**
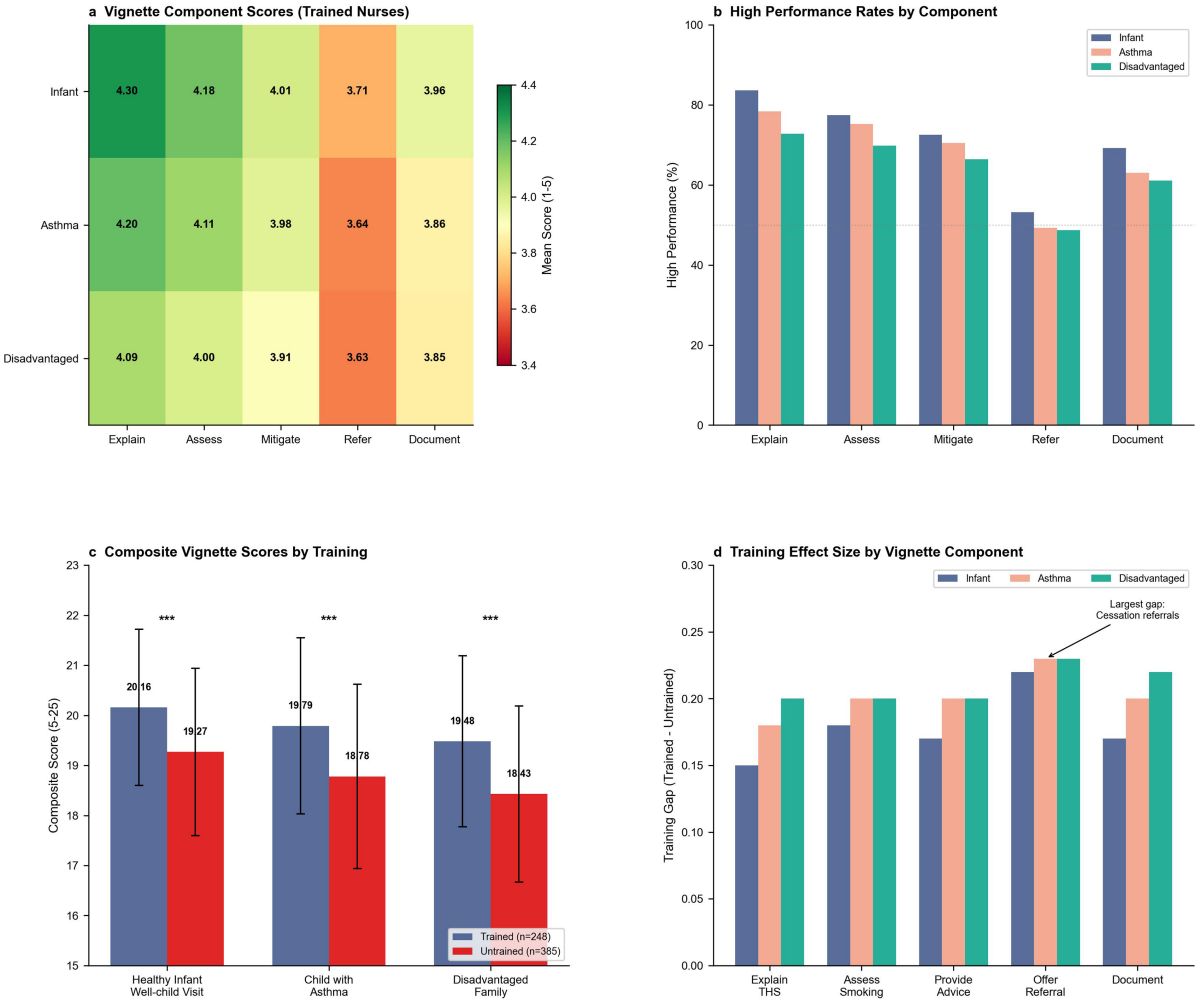
Clinical vignette performance and training impact across scenarios. **(a)** Heatmap showing mean component scores for five counseling behaviors across three clinical scenarios (Infant, Asthma, Disadvantaged) among trained nurses. Color intensity indicates performance level (scale: 3.4–4.4). **(b)** Percentage of nurses achieving high performance (score ≥4) by component and scenario across three patient populations. **(c)** Composite vignette scores (mean ± SD) by training status across three scenarios. ***p < 0.001. **(d)** Training effect sizes (mean difference: Trained minus Untrained) by component and scenario. Largest effect indicated by annotation.

Applied counseling performance in standardized clinical scenarios demonstrated consistent training effects across all three vignettes and all five counseling components. For the healthy infant well-child visit scenario, trained nurses achieved a mean composite score of 20.16 ± 1.56 out of 25 possible points, compared to 19.27 ± 1.67 for untrained nurses (*p* < 0.001). Similar patterns emerged for the asthma exacerbation scenario (trained: 19.79 ± 1.76, untrained: 18.78 ± 1.84, *p* < 0.001) and the socioeconomically disadvantaged family scenario (trained: 19.48 ± 1.71, untrained: 18.43 ± 1.76, *p* < 0.001), with trained nurses consistently outperforming untrained colleagues by approximately 0.9–1.0 points per vignette (Figure 4c). Across all three vignettes, a consistent performance hierarchy emerged among trained nurses: explaining THS concepts received the highest scores, followed by assessing smoking practices, providing mitigation advice, offering cessation referrals, and documentation (full component-level data in Table 5 and Figure 4a). Cessation referral performance was markedly lower than explanation across all scenarios (high-performance rates: 48.7–53.2% vs. 72.8–83.6%, respectively; Figure 4b). Training effect sizes were largest for cessation referrals and documentation (mean differences 0.17–0.23 points) and smallest for THS explanation (0.15–0.20 points; Figure 4d).

**Table 5:** Clinical vignette component performance across three scenarios (*N* = 633).

Vignette and component	Mean ± SD	High performance no. (%)	Trained	Untrained	Urban	Rural	Bachelor+	Diploma	*p*
Infant: Explain thirdhand smoke concept	4.21 ± 0.73	529 (83.6)	4.3	4.15	4.23	4.11	4.22	4.14	0.012
Infant: Assess home and car smoking	4.07 ± 0.77	490 (77.4)	4.18	4	4.09	3.99	4.09	3.99	0.006
Infant: Provide mitigation advice	3.91 ± 0.79	459 (72.5)	4.01	3.84	3.93	3.82	3.92	3.86	0.015
Infant: Offer cessation referral	3.58 ± 0.83	337 (53.2)	3.71	3.49	3.6	3.48	3.6	3.48	0.002
Infant: Document in medical record	3.86 ± 0.77	438 (69.2)	3.96	3.79	3.88	3.78	3.87	3.81	0.009
Asthma: Explain thirdhand smoke concept	4.09 ± 0.79	496 (78.4)	4.2	4.02	4.11	3.99	4.11	4	0.007
Asthma: Assess home and car smoking	3.99 ± 0.79	476 (75.2)	4.11	3.91	4.01	3.89	4	3.94	0.004
Asthma: Provide mitigation advice	3.86 ± 0.81	446 (70.5)	3.98	3.78	3.88	3.76	3.87	3.8	0.005
Asthma: Offer cessation referral	3.50 ± 0.84	312 (49.3)	3.64	3.41	3.52	3.4	3.52	3.42	0.001
Asthma: Document in medical record	3.74 ± 0.81	399 (63.0)	3.86	3.66	3.76	3.66	3.75	3.69	0.005
Disadvantaged: Explain thirdhand smoke concept	3.97 ± 0.80	461 (72.8)	4.09	3.89	3.99	3.87	3.98	3.91	0.004
Disadvantaged: Assess home and car smoking	3.88 ± 0.79	442 (69.8)	4	3.8	3.9	3.78	3.89	3.83	0.003
Disadvantaged: Provide mitigation advice	3.79 ± 0.82	420 (66.4)	3.91	3.71	3.81	3.69	3.8	3.74	0.005
Disadvantaged: Offer cessation referral	3.49 ± 0.85	308 (48.7)	3.63	3.4	3.51	3.39	3.51	3.41	0.001
Disadvantaged: document in medical record	3.72 ± 0.85	387 (61.1)	3.85	3.63	3.74	3.64	3.73	3.67	0.003

Vignettes presented realistic clinical scenarios requiring thirdhand smoke counseling. Components rated on 5-point scale (1 = would definitely not do to 5 = would definitely do). High performance defined as rating ≥4. Infant refers to healthy infant at routine well-child visit; Asthma, child with asthma exacerbation; Disadvantaged, socioeconomically disadvantaged family. Bachelor+ includes Bachelor, Master, and Doctorate degrees. *p* values from Mann–Whitney U tests comparing trained vs untrained nurses.

### 3.6 Implementation context: barriers and facilitators

Perceived barriers to THS counseling implementation were endorsed at high rates across the sample. Lack of training or materials represented the most frequently cited barrier, with 67.3% of nurses rating this as a substantial obstacle (mean score: 3.87 ± 0.86 on a 1–5 scale). Insufficient time during clinical encounters was endorsed by 58.1% (mean: 3.68 ± 0.89), absence of clear protocols by 51.2% (mean: 3.56 ± 0.88), and fear of offending parents by 38.4% (mean: 3.20 ± 0.96). Notably, these barrier ratings did not differ significantly between trained and untrained nurses, suggesting that training alone does not eliminate structural and interpersonal obstacles (Table 6).

**Table 6:** Barriers, facilitators, and system factors stratified by nurse characteristics (*N* = 633).

Item	Mean ± SD or no. (%)	High endorsement no. (%)	Trained	Untrained	Urban	Rural	Bachelor+	Diploma	*p*
Insufficient time during encounters	3.68 ± 0.89	368 (58.1)	3.66	3.69	3.72	3.46	3.67	3.73	0.658
Lack of training or materials	3.87 ± 0.86	426 (67.3)	3.84	3.89	3.79	4.15	3.86	3.94	0.451
Fear of offending parents	3.20 ± 0.96	243 (38.4)	3.18	3.21	3.14	3.41	3.19	3.26	0.669
Absence of protocols	3.56 ± 0.88	324 (51.2)	3.53	3.58	3.48	3.87	3.55	3.62	0.469
Counseling scripts available	4.18 ± 0.79	505 (79.8)	4.19	4.17	4.2	4.07	4.19	4.11	0.727
Clear referral pathways	4.01 ± 0.83	469 (74.1)	4.06	3.98	4.03	3.92	4.02	3.96	0.279
Leadership emphasis	3.98 ± 0.81	462 (73.0)	4.01	3.96	4	3.88	3.99	3.93	0.493
Peer champion support	3.86 ± 0.90	423 (66.8)	3.9	3.83	3.88	3.76	3.87	3.8	0.391
Routine screening protocols	262 (41.4)	—	109 (44.0)	153 (39.7)	171 (43.0)	33 (38.8)	—	—	0.084
EMR documentation required	294 (46.4)	—	123 (49.6)	171 (44.4)	191 (48.0)	36 (42.4)	—	—	0.021
Referral resources accessible	270 (42.7)	—	114 (46.0)	156 (40.5)	177 (44.5)	33 (38.8)	—	—	0.021
In competency or QI metrics	231 (36.5)	—	99 (39.9)	132 (34.3)	152 (38.2)	28 (32.9)	—	—	0.021
Leadership support score	3.79 ± 0.90	—	3.81	3.78	3.81	3.71	—	—	0.472

Barriers and facilitators rated on 5-point scale (1 = strongly disagree to 5 = strongly agree). High endorsement defined as rating ≥4. System factors reported as “Yes” responses for categorical items (routine screening, EMR requirements, referral resources, QI inclusion) or mean score for leadership support. Bachelor+ includes Bachelor, Master, and Doctorate degrees. *p* values from Mann–Whitney U tests or χ^2^ tests comparing trained vs untrained nurses. EMR indicates electronic medical record; QI, quality improvement.

Facilitators were perceived as more readily available than barriers were perceived as severe. Counseling scripts were reported as available by 79.8% of nurses (mean: 4.18 ± 0.79), leadership emphasis on tobacco control by 73.0% (mean: 3.98 ± 0.81), clear referral pathways by 74.1% (mean: 4.01 ± 0.83), and peer champion support by 66.8% (mean: 3.86 ± 0.90). Again, facilitator ratings showed no significant differences between trained and untrained groups (Figure 3d).

System-level implementation factors revealed moderate institutional support for THS counseling. Routine screening protocols for tobacco exposure existed for 41.4% of the sample, EMR documentation requirements for 46.4%, accessible referral resources for 42.7%, and inclusion of tobacco counseling in competency or quality improvement metrics for 36.5%. Trained nurses reported significantly higher access to EMR documentation requirements (49.6% vs. 44.4%, *p* = 0.021), referral resources (46.0% vs. 40.5%, p = 0.021), and quality improvement inclusion (39.9% vs. 34.3%, *p* = 0.021; Table 6).

## 4 Discussion

Among 633 Chinese pediatric nurses, formal tobacco-control training was the strongest correlate of counseling practice scores (*β* = 2.23, accounting for 6.8% of variance in an observational, cross-sectional model; causal inference is not warranted), while knowledge, attitudes, and self-efficacy collectively added only 0.4% explanatory power—a finding that fundamentally challenges cognitive mediation models underlying most health professional education. Even among trained nurses, absolute counseling rates remained suboptimal: only 51.6% consistently explained THS concepts, 47.6% assessed household smoking, and 44.4% offered cessation referrals. Vignette performance revealed a troubling equity gradient, with composite scores declining from 20.16 ± 1.56 for healthy infant visits to 18.43 ± 1.76 for disadvantaged families (*p* < 0.001)—precisely the population facing 40–60% higher household smoking rates. These findings emerge within China’s tobacco epidemic, where 300 million smokers and 50% male smoking prevalence create pervasive childhood exposure, yet cultural norms around filial piety and multi-generational households complicate intervention delivery (29, 31, 52, 53).

Our training coefficient (*β* = 2.23, 95% CI: 1.56–2.89, *p* < 0.001) corresponds to a standardized effect size of 0.39 SD (calculated as unstandardized coefficient divided by outcome SD: 2.23/5.72 = 0.39), indicating a moderate effect magnitude by conventional benchmarks. Carson et al.’s meta-analysis of 47 tobacco control training studies found effect sizes of 0.3–0.8 SD (54), aligning with our 0.39 SD estimate. However, it should be noted that systematic reviews have identified substantial heterogeneity in training effectiveness (I^2^ = 68%), with several null findings in real-world implementation contexts, particularly when training is brief (<4 h) or lacks ongoing reinforcement (55, 56). Sequential model comparison illustrates the dominance of training. Model 1 (demographics only) explained virtually no variance (R^2^ = 0.002). Adding training in Model 2 increased R^2^ to 0.070 (ΔR^2^ = 0.068). Adding all cognitive factors in Model 3 raised R^2^ only marginally to 0.074 (ΔR^2^ = 0.004)—a 17-fold difference in explanatory power. This pattern sharply diverges from Godin et al.’s systematic review of 76 healthcare professional behavior studies, where cognitive factors typically explained 30–40% of behavioral variance (57, 58). Our findings suggest that in resource-constrained, high-pressure clinical environments, training’s impact operates through mechanisms other than conscious cognitive processing.

Three explanations warrant consideration: (1) procedural knowledge and behavioral scripts not captured by declarative knowledge assessments—Carson demonstrated that skill-practice training produces superior outcomes even with equivalent declarative knowledge gains (59); (2) institutional legitimacy signaling that reduces role boundary uncertainty—qualitative research shows nurses often possess adequate knowledge but refrain from counseling due to unclear professional mandates (60, 61); and (3) workflow integration strategies that bypass conscious knowledge retrieval during time-pressured encounters. Supporting the legitimacy hypothesis, trained nurses demonstrated significantly higher policy awareness (57.7% versus 41.3%, *p* < 0.001), suggesting training confers not just skills but professional authorization to engage in tobacco counseling.

Drawing on the broader literature rather than the present data, evidence-based training design has been proposed to require 10–20 h across multiple sessions with deliberate practice and feedback, authentic scenario simulation reflecting clinical complexity, and ongoing reinforcement through booster sessions and peer networks—components that were not assessed in this study but whose presence or absence may partly explain why current training, though associated with higher practice scores, did not fully correct misconceptions or eliminate equity gradients (62–64). McGaghie et al.’s meta-analysis of simulation-based medical education demonstrated that deliberate practice with feedback produces effect sizes of 0.71–1.20 SD, nearly double our observed 0.4 SD, suggesting substantial room for training optimization (62). Current training approaches, while superior to none, appear insufficient for sustained, comprehensive implementation.

In the present study, Chinese nurses demonstrated strong factual knowledge—83.7% recognized residue persistence, 79.5% knew THS persists weeks-to-months—comparable to US/European benchmarks of 65–85% (65, 66). Trained nurses outperformed untrained colleagues by 7.5 percentage points on residue persistence (88.3% vs. 80.8%, *p* = 0.011), indicating that current training does improve declarative knowledge even if this does not translate proportionally to practice (Table 7).

**Table 7:** Composite vignette scores by nurse characteristics (*N* = 633).

Vignette scenario	Overall	Trained	Untrained	Urban	Rural	Bachelor+	Diploma	*p* value
	Mean ± SD	Mean ± SD	Mean ± SD	Mean	Mean	Mean	Mean	
Healthy infant at routine well-child visit	19.63 ± 1.68	20.16 ± 1.56	19.27 ± 1.67	19.73	19.18	19.7	19.28	<0.001
Child with asthma exacerbation	19.18 ± 1.86	19.79 ± 1.76	18.78 ± 1.84	19.28	18.7	19.25	18.85	<0.001
Socioeconomically disadvantaged family	18.85 ± 1.80	19.48 ± 1.71	18.43 ± 1.76	18.95	18.37	18.91	18.56	<0.001

Composite scores calculated as sum of 5 counseling components per vignette (range, 5–25). Higher scores indicate greater likelihood of comprehensive thirdhand smoke counseling. Bachelor+ includes Bachelor, Master, and Doctorate degrees. *p* values from Mann–Whitney U tests comparing trained vs untrained nurses.

This approximately 45–50 percentage point gap between factual knowledge and misconception rejection represents a more severe deficit. Drehmer’s US pediatrician study found 89% knew THS persists but only 41% rejected ventilation strategies—a 48-point gap comparable to ours (67, 68). However, Haardörfer’s population-based US survey found only 23% of adults correctly rejected the “no odor, no exposure” misconception, making our nurses’ 34.4% accuracy significantly better than general public knowledge yet far below the 80% + standard expected for healthcare professionals (65, 66). Training improved misconception recognition modestly: trained nurses scored 11.0 percentage points higher on the odor misconception (41.1% versus 30.1%, *p* = 0.005) and 8.3 points higher on balcony smoking (41.5% versus 33.2%, *p* = 0.034), but absolute performance remained unacceptably low even among trained providers.

These misconceptions carry serious health consequences: nicotine reactions with nitrous acid generate carcinogenic TSNAs (NNK, NNA) persist for months; cleaning may paradoxically increase exposure through particulate aerosolization; and children’s 2–3 × higher respiratory rates per kilogram plus immature hepatic detoxification create disproportionate vulnerability (1, 7, 18, 69–71). Sleiman et al. demonstrated that surface-mediated nicotine reactions continue for weeks, generating NNK concentrations reaching 10–50 ng/surface—levels sufficient to induce DNA damage in cellular models (7, 8). Martins-Green’s animal studies revealed that even third-generation exposure (no direct smoke contact) induced 30–40% increases in inflammatory markers and 25% reductions in wound healing rates, establishing biological plausibility for health effects (8).

From a cognitive psychology perspective, these beliefs represent availability heuristics reinforced by visible smoke dispersal and culturally entrenched spatial separation practices in Chinese households (72). Simple factual instruction proves insufficient; misconception correction requires explicit confrontation of false beliefs through cognitive conflict strategies. Xiao et al.’s national Chinese survey found 67% of smokers believed smoking near windows or on balconies adequately protects children—a misconception so deeply embedded that even healthcare professionals struggle to overcome it (73).

The weak association between knowledge scores and counseling practices (*β* = 0.14, *p* = 0.217) may reflect our measure’s emphasis on declarative factual recall rather than conditional procedural knowledge of when and how to apply THS counseling in specific clinical contexts. However, this interpretation is speculative, as we did not independently validate that our knowledge items assess clinically relevant cognitive constructs. The poor internal consistency (*α* = 0.060) alternatively suggests the items may simply be measuring unrelated factual domains without coherent underlying structure. Additional research with validated knowledge instruments explicitly designed to predict counseling behavior is needed to adjudicate between these interpretations. Clinical communication research confirms declarative knowledge correlates weakly (*r* = 0.15–0.25) with performance, while procedural knowledge of communication strategies demonstrates stronger prediction (*r* = 0.40–0.55) (12, 15, 56). This distinction may explain why our knowledge scale demonstrated low internal consistency (Cronbach’s α = 0.060), suggesting these items measure multiple independent knowledge domains rather than a single construct.

The observed null association between self-efficacy and practice (*β* = 0.00, *p* = 0.991) is noteworthy but should not be interpreted as evidence that self-efficacy is irrelevant to counseling behavior. Global self-efficacy ratings assessed without scenario-specific anchoring are known to predict behavior less reliably than task-specific measures, and attenuation due to measurement imprecision cannot be excluded as an explanation for this finding. However, this aligns with evidence that global self-efficacy ratings predict behavior less accurately than task-specific assessments anchored to concrete scenarios, and that self-reported confidence often reflects illusory superiority rather than competence (1, 13, 14). Bandura’s Social Cognitive Theory predicts self-efficacy should mediate training effects on behavior, yet our data show training effects persist undiminished even after controlling for self-efficacy—a theoretical anomaly requiring explanation (55, 56). Moreover, with 58.1% reporting insufficient time and 51.2% lacking protocols, structural constraints may render individual readiness statistically irrelevant—even highly confident, motivated nurses cannot implement behaviors without institutional infrastructure. This interpretation gains support from practice setting analysis: urban nurses reported marginally higher practice scores than rural nurses (40.7% versus 37.6% optimal practice rates), but this 3.1-point difference was not statistically significant (*p* = 0.156), suggesting that system-level factors overwhelm individual or setting characteristics. Similarly, years of experience showed minimal gradients: nurses with ≥10 years achieved only 2.8 percentage points higher optimal practice rates than those with <5 years, despite presumably greater confidence and expertise.

Sarna et al.’s survey of 1,425 US nurses found that perceived organizational support predicted smoking cessation counseling more strongly (OR = 2.8) than self-efficacy (OR = 1.4), corroborating our finding that structural factors may dominate individual psychological readiness (60). The absence of significant associations between our barrier and facilitator measures with practice scores challenges this interpretation but may reflect measurement limitations rather than true null effects.

Vignette analysis revealed three critical patterns: (1) consistent training advantages across all scenarios (0.89–1.05 point differences, all *p* < 0.001); (2) universal weakness in cessation referrals (53.2% high performance for infant scenario versus 83.6% for explaining THS); and (3) declining performance for disadvantaged families despite their 40–60% higher household smoking exposure burden (35, 61, 74, 75). Breaking down vignette components reveals the performance hierarchy: explanation of THS concepts achieved highest scores (infant: 4.30 ± 0.73, asthma: 4.20 ± 0.79, disadvantaged: 4.09 ± 0.80), followed by assessment of smoking practices (infant: 4.18 ± 0.77, asthma: 4.11 ± 0.79, disadvantaged: 4.00 ± 0.79), then mitigation advice (range: 3.91–4.01), with cessation referrals (range: 3.58–3.71) and documentation (range: 3.86–3.96) consistently lowest. Training effect sizes varied by component: cessation referrals showed largest training gaps (0.17–0.23 points), followed by documentation (0.17–0.20 points) and assessment (0.18–0.20 points), with smaller but significant effects for explanation (0.15–0.20 points) and mitigation advice (0.17–0.20 points).

The 1.73-point decline in composite scores from healthy infant (20.16 ± 1.56) to disadvantaged family scenarios (18.43 ± 1.76) suggests unconscious rationing based on perceived receptivity or deservingness—a troubling equity gap requiring explicit attention in training curricula. This differential compounds existing disparities: Li et al.’s national Chinese survey documented smoking prevalence of 64.8% among low-education males versus 39.2% among college-educated males (74). Moreover, low-income families report 45% less access to smoking cessation resources and 38% lower cessation success rates, creating a vicious cycle of exposure and limited intervention (35, 74).

Cessation referral weakness (48.7–53.2% high performance across scenarios) replicates broader smoking cessation research showing providers’ greater comfort with education than behavior change facilitation. Singh et al.’s survey of 412 healthcare providers found only 37% felt comfortable making cessation referrals versus 76% comfortable providing information—a 39-point gap nearly identical to our 30-point gap between explanation (83.6%) and referrals (53.2%) (76). Referrals demand resource navigation, readiness assessment, and collaborative goal-setting—complex skills requiring supervised practice, not didactic instruction. Our finding that trained nurses outperformed untrained colleagues by only 10.1 percentage points on cessation referrals (44.4% versus 34.3%) versus 13.2 points on THS explanation (51.6% versus 38.4%) suggests current training inadequately addresses this critical skill gap.

Reported facilitators were not significantly associated with practice scores (*β* = 0.10, *p* = 0.297); likewise, barriers did not reach significance (*β* = −0.11, *p* = 0.188). While this pattern may suggest that nominal resource availability does not translate to practice, it is equally plausible that the brief, four-item barrier and facilitator scales lacked sufficient depth or psychometric precision to capture the implementation constructs they were intended to measure. These null findings should therefore be regarded as hypothesis-generating rather than as confirmation that environmental context is unimportant to THS counseling implementation (29, 36, 39, 42, 77). Three interpretations merit consideration; First, availability does not equal usability. Guideline implementation research demonstrates that nominal access predicts adoption poorly compared to workflow integration and tool usability measures (66, 67, 77, 78). Nurses may have theoretical access to scripts too cumbersome for 8-min encounters or referral pathways requiring 15-min phone navigation. Supporting this interpretation, practice scores showed minimal variation across settings despite presumably different resource availability: general pediatrics (40.2% optimal practice), NICU (40.5%), PICU (39.8%), outpatient (38.9%), and emergency (37.2%) demonstrated a narrow 3.3-point range, suggesting systemic constraints operate uniformly across clinical contexts.

Second, the Consolidated Framework for Implementation Research (CFIR) identifies five implementation domains: intervention characteristics, outer setting, inner setting, individual characteristics, and process (79). Our measurement emphasized inner setting and individual factors, largely neglecting intervention design quality and active implementation strategies. Complex behavioral interventions require active facilitation—not passive resource provision—through implementation champions, ongoing coaching, and iterative adaptation (41, 53, 80). Damschroder’s CFIR validation study found that “implementation climate” (active strategies) predicted adoption (OR = 4.2) far more strongly than “available resources” (OR = 1.6), suggesting our barrier/facilitator measures captured the wrong constructs (79).

Notably, trained and untrained nurses reported similar barrier perceptions: approximately 67% of both groups (67% trained, 67% untrained) cited inadequate training/materials (*p* = 0.45). While these differences did not reach statistical significance, we cannot conclude equivalence without formal equivalence testing. The similar barrier perceptions despite training may indicate that training provides skills without addressing systemic structural obstacles, or may reflect limitations in our barrier measurement approach. This explains training’s practice effects despite unchanged barrier perceptions. However, trained nurses did report significantly higher access to system-level supports: EMR documentation requirements (49.6% versus 44.4%, *p* = 0.021), referral resources (46.0% versus 40.5%, *p* = 0.021), and QI metric inclusion (39.9% versus 34.3%, *p* = 0.021). This 5–6 percentage point advantage suggests training may cluster within better-resourced units or that trained nurses receive preferential institutional selection bias potentially inflating training effects.

Although not directly tested in the present study, the pattern of findings—training as the dominant correlation of practice, persistent misconception gaps despite training, and an equity gradient in vignette performance—suggests several directions for future intervention research that are grounded in the broader implementation science and tobacco control education literature. These should be regarded as evidence-informed hypotheses requiring prospective evaluation rather than conclusions supported by the current data. First, future trials might evaluate whether training redesign from brief didactic workshops to multi-session programs (proposed minimum 12–16 h) emphasizing deliberate practice with standardized patients, explicit misconception confrontation through cognitive conflict strategies, and supervised cessation referral skill-building produces practice improvements beyond those associated with current training formats. Second, intervention studies could examine whether explicit health equity competency development addressing unconscious biases in counseling allocation reduces the vignette-based performance gap observed for disadvantaged families in this study. Third, workflow redesign studies—including EMR hard-stops triggered by tobacco exposure documentation and integrated referral pathways—could test whether translating theoretically available resources into embedded workflow tools improves practice rates. Fourth, policy evaluation research could examine whether national-level mandates requiring THS screening in all pediatric encounters reduce the inter-institutional variation in training access and counseling rates implied by the present findings.

Comparative international evidence supports these recommendations: Australia’s “Smoking Cessation in Hospitals” program increased nursing counseling rates from 41 to 78% through EHR integration, champion networks, and monthly audit-feedback—precisely the active implementation strategies absent in our study setting (81, 82). Given China’s 300 million smokers and pervasive childhood household exposure affecting an estimated 180 million children (54% of the pediatric population), pediatric nurse-delivered THS counseling represents a scalable, high-impact intervention—if implementation barriers can be systematically addressed. At baseline optimal practice rates of 40%, our 633-nurse sample delivers comprehensive THS counseling to approximately 10,000 pediatric encounters annually. Increasing optimal practice to 75% through enhanced training and system supports would potentially reach an additional 14,000 families yearly, with multiplicative effects as household smoking reduction protects multiple children per family.

Several limitations should be considered, ordered by their likely impact on interpretation.

First, the cross-sectional design precludes causal inference about training effects. Trained and untrained nurses may differ systematically in motivation, competence, or institutional support—unmeasured confounds that could inflate the observed training coefficient. The finding that trained nurses reported 5–6 percentage points higher access to EMR requirements and referral resources (*p* = 0.021) further suggests selection bias through clustering of training in better-resourced units. Prospective randomized or quasi-experimental designs are needed to establish whether training itself drives practice improvement. Second, poor psychometric reliability limits confidence in regression-based estimates. The knowledge composite (Cronbach’s *α* = 0.060) and practice composite (Cronbach’s α = 0.249) both fall below acceptable thresholds, introducing attenuation bias that deflates coefficients toward the null; true associations may be larger than observed. The knowledge scale also emphasizes declarative recall over procedural and conditional knowledge more directly relevant to counseling performance, which may further explain the weak knowledge–practice association. Future studies should develop and validate psychometrically sound THS-specific instruments before inferential use. Third, the single-center tertiary hospital design limits generalizability to secondary hospitals, rural facilities, and other national or international contexts. Fourth, self-reported practices are susceptible to social desirability bias and likely overestimate counseling frequencies. Fifth, vignette-based assessment captures behavioral intentions rather than actual clinical performance; research suggests only moderate correlations (*r* = 0.45–0.60) between vignette responses and observed practice. Sixth, training was assessed as a binary self-report variable without measurement of content, duration, or pedagogical quality, preventing identification of which training components drive the association. Finally, the absence of patient-level outcomes—parental behavior change, household smoke-free policy adoption, or child biomarker data—leaves uncertain whether higher practice scores translate to meaningful health benefits. These limitations indicate that findings should be interpreted as hypothesis-generating, warranting prospective trials with objective behavioral and biomarker outcomes.

## 5 Conclusion

In this cross-sectional study of 633 Chinese pediatric nurses, training was the strongest correlate of THS counseling practices, yet absolute performance remained suboptimal even among trained nurses: key misconceptions persisted, and counseling quality declined for the socioeconomically disadvantaged families facing the greatest household smoking burden. The gap between high reported resource availability and suboptimal practice points to systemic implementation failures—resources exist nominally but lack the workflow integration needed for routine clinical use. These findings are associational, and psychometric limitations of the instruments warrant caution in interpreting effect magnitude. The results nonetheless generate a clear research agenda: prospective trials are needed to test whether skills-based training redesign, workflow-embedded decision supports, and equity-focused competency development can convert the observed training–practice association into genuine, sustained improvements in child health. In China’s high-burden tobacco environment, protecting children from thirdhand smoke requires more than knowledge—it requires system-level structures that embed counseling into routine pediatric care.

## Data Availability

The raw data supporting the conclusions of this article will be made available by the authors, without undue reservation.
